# Determinants of outpatient healthcare-seeking behaviors among the rural poor affected by chronic conditions in India: a population-based cross-sectional study in seven states

**DOI:** 10.1080/16549716.2025.2480413

**Published:** 2025-04-14

**Authors:** David Grossmann, Swati Srivastava, Volker Winkler, Stephan Brenner, Keerti Jain Gupta, Amit Paliwal, Kavita Singh, Manuela De Allegri

**Affiliations:** a Heidelberg Institute of Global Health, Heidelberg University Hospital and Medical Faculty, Heidelberg, Germany; b Indo German Programme on Universal Health Coverage (IGUHC), Deutsche Gesellschaft für Internationale Zusammenarbeit (GIZ) GmbH, New Delhi, India; c Centre for Chronic Conditions and Injuries, Public Health Foundations of India, Gurugram, India

**Keywords:** Quality of Care for Chronic Conditions, Health services, primary health care, non-communicable diseases, multinomial logistic regression, socio-economic, low-and-middle-income countries, private sector, public sector, treatment, India

## Abstract

**Background:**

A rising burden of chronic non-communicable diseases (CNCDs) increases demand for outpatient healthcare. Yet, evidence on preferences and barriers to healthcare services for India's most disadvantaged population, the target of India's largest public health insurance scheme (PM-JAY), is lacking.

**Objective:**

We explore determinants of outpatient healthcare-seeking behavior among PM-JAY eligible individuals with CNCDs in rural areas of seven states.

**Methods:**

Using cross-sectional data from a household survey (conducted between November 2019 and March 2020), we employed multilevel multinomial logistic regression to identify factors associated with seeking care from informal (home treatment, pharmacies, traditional healers), formal public, or formal private providers, compared with no care. Anderson's behavioral model informed the selection of independent variables.

**Results:**

Of 51,820 individuals, 5,061 (9.8%) reported a chronic condition. Despite their disease, 1,168 (23.1%) reported not using regular outpatient care. Another 2,421 individuals (48.0%) used formal private, 922 (18.3%) used formal public, and 535 (10.6%) used informal care. Predictors of formal private care were higher socioeconomic status (RRR = 2.441, 95% CI [1.61, 3.70]) and health insurance coverage (RRR = 1.478, 95% CI [1.12, 1.95]). Residents of Tamil Nadu, Kerala, and Gujarat were more likely to use formal public care (RRR = 23.915, 95% CI [9.01, 63.44]). Suffering from Major CNCDs or experiencing limitations in daily activities increased the probability of using healthcare across all options.

**Conclusion:**

Future research should explore the reasons for non-utilization of chronic care and the preference for private providers. Policies to enhance public healthcare utilization and expand insurance for outpatient care could improve access and reduce health inequities.

## Paper Context

- **Main findings**: Among India's rural populations, outpatient health service use for chronic non-communicable diseases is largely concentrated in the private sector and primarily determined by a patient's socioeconomic status.
- **Added knowledge**: We shed light on healthcare-seeking behavior for socioeconomically disadvantaged individuals eligible for India's largest publicly funded health insurance system and highlight substantial interstate disparities in public healthcare utilization.
- **Global health impact for policy and action**: To meet the 2030 Sustainable Development Goals, India's public healthcare system must be strengthened to provide equitable access to effective chronic disease treatment, improve health outcomes, and tackle the growing global burden of chronic non-communicable diseases.

## Background

Chronic non-communicable diseases (CNCDs), including cardiovascular diseases, diabetes, cancer, and respiratory illnesses, pose a growing global health challenge, particularly in low- and middle-income countries (LMICs) [1]. They cause 71% of global deaths, with 77% occurring in LMICs, many of them premature and preventable [2]. This challenges healthcare systems in resource-constrained settings, which are typically designed for acute, episodic care rather than chronic disease management [3]. Managing chronic diseases requires continuous, integrated care, regular follow-ups, and sustained medication regimens [4], ideally at subclinical stages before complications necessitate hospitalization. However, LMICs often face healthcare infrastructure limitations, workforce shortages, low insurance coverage [5], and poor disease awareness, all of which lead to delayed diagnosis and treatment [6]. The economic burden of chronic diseases further exacerbates poverty through high out-of-pocket expenditures and loss of productivity, disproportionately affecting poor and rural populations [7,8].

Comprehensive outpatient care at the primary healthcare level is a cornerstone to control CNCDs and reduce premature death [9]. While addressing CNCDs is recognized as crucial for achieving universal health coverage (UHC) and the 2030 Sustainable Development Goals (SDGs) [7], disease control rates in LMICs remain poor [10], and access to services is highly unequal [5]. Utilization of outpatient care is shaped by barriers on both the demand and supply side, many of which remain under-researched [11]. Studies from Sub-Saharan Africa [12,13] and Latin America [14] document the role of age, gender, illness severity, and socioeconomic status in healthcare-seeking for CNCDs. In Southeast Asia, health insurance, multimorbidity, a higher education level, and health beliefs affect service utilization [15–17]. Across all these regions, the availability of services, staff, medicines, and facility accessibility (including proximity and operating hours) are key determinants [14,18]. In many LMICs, patients often switch between public and private providers based on perceived quality, availability, and cost [18]. Despite growing evidence, comprehensive quantitative data around determinants of healthcare-seeking behavior for CNCDs in LMIC remain lacking [18], particularly for regular outpatient care among poor, marginalized populations. This knowledge gap on barriers to care hinders the development of effective interventions and policies tailored to their specific needs.

In India, CNCDs account for 58% of disability-adjusted life years (DALYs) and 65% of the overall mortality [19]. Understanding outpatient healthcare-seeking behavior in India, given its size and diversity, provides valuable insights for improving CNCD care in low-resource settings. Prior studies from India show increased outpatient care use among those with higher education [20] and multimorbidity [21,22], with an overall preference for private providers. However, the poor and elderly rely more on public health [23–25]. Existing studies are often limited by scope, focusing on specific chronic conditions (such as diabetes or hypertension) [24,26], sub-populations (for example, elderly, widows, or aboriginals) [20,22,27], or regions, and, therefore, failed to account for differences across India's heterogeneous states [21,23,25,28,29]. Reliable data on factors influencing outpatient healthcare utilization for chronic conditions among the most marginalized rural poor remain scarce. This study addresses this knowledge gap by analyzing outpatient healthcare use and its determinants among the rural poor in seven Indian states.

## Methods

### Study setting and sampling

This study used data from a cross-sectional household survey conducted between November 2019 and March 2020 in rural areas of seven Indian states: Bihar, Chhattisgarh, Gujarat, Kerala, Meghalaya, Tamil Nadu, and Uttar Pradesh. The survey evaluated the implementation and early effects of ‘Pradhan Mantri Jan Arogya Yojana’ (PM-JAY), the national health protection scheme launched in 2018 as part of the Ayushman Bharat healthcare reform. PM-JAY subsumes previously existing publicly funded health insurance schemes (PFHI), offering extended insurance coverage for secondary and tertiary care hospitalization to approximately 500 million Indians, including the poorest households [30]. Further details about the parent evaluation study of PM-JAY are described elsewhere [31].

**Figure 1.**
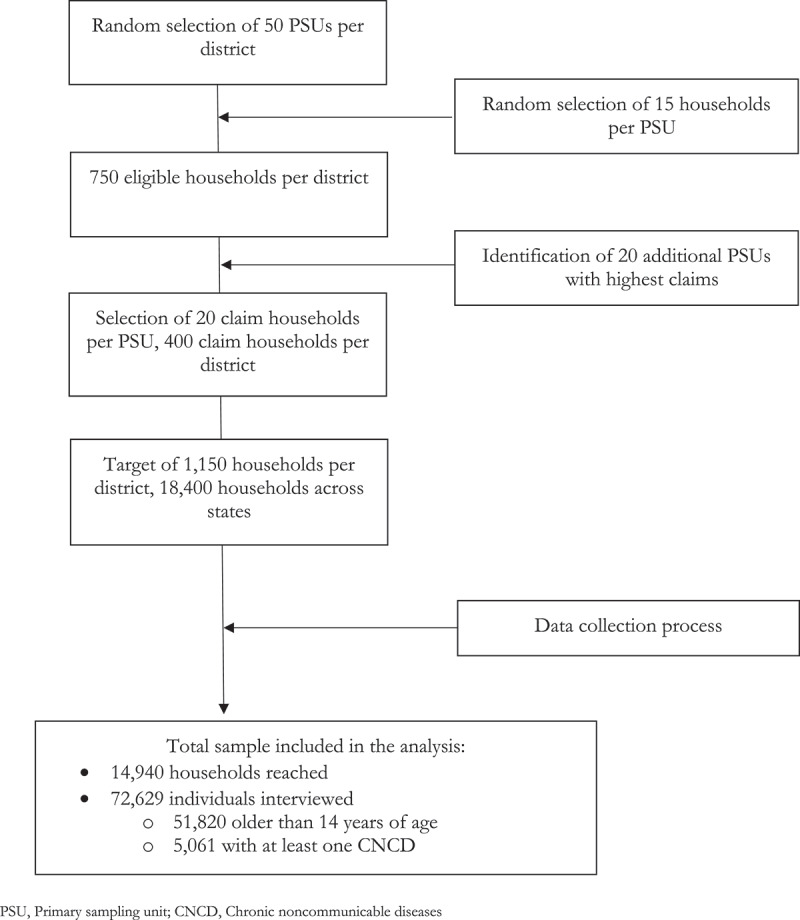
Sampling strategy and data collection.

Briefly, study states were purposefully selected based on geographical diversity, the presence of previous PFHI schemes, and the state's implementation approach to PM-JAY. Two districts per state (three for Bihar and Uttar Pradesh) were purposively selected based on healthcare performance within states. Administrative blocks within each rural village functioned as the primary sampling unit (PSU). The sampling strategy followed a three-step approach (Figure 1). First, for each district, 50 representative PSUs were randomly selected using information from the 2011 Socioeconomic and Caste Census (SECC), a nationwide survey conducted to identify social welfare program beneficiaries. Next, 15 households per PSU were randomly selected based on SECC data, giving 750 households per district. Lastly, the parent study identified an additional 20 PSUs among those with the highest PM-JAY insurance claims for hospitalizations, to ensure sufficient eligible households with PM-JAY claims, with 20 households chosen from each PSU. This resulted in a total target sample of 1,150 households per district and 18,400 households across states. As the 2020 COVID-19 pandemic disrupted the sampling process in Kerala, the effective sample consists of 72,629 individuals from 14,940 households. After excluding children under 14 years to focus on CNCDs in adolescents and adults, the final sample included 51,820 individuals, distributed across the following states: Bihar (10,383), Chhattisgarh (7,209), Gujarat (7,640), Meghalaya (7,109), Tamil Nadu (4,752), Kerala (2,758), and Uttar Pradesh (11,969).

### Data collection

A structured questionnaire collected self-reported information on household socio-demographics, economic status, acute and chronic health conditions, healthcare use, out-of-pocket spending on health, and awareness of PM-JAY. The survey was administered in local languages using computer-assisted personal interviewing. For this study, data were drawn from sections on household composition, socioeconomic background, chronic illness reporting, and outpatient health service use. All data were collected individually for each household member, with an identifier to determine the individual's household affiliation. Health service use was assessed over a 30-day recall period.

### Variables and their measurements

Table 1 displays all variables and their measurements included in this study. Our outcome variable is the type of outpatient healthcare provider used by individuals with a chronic condition in the past 30 days. We categorized health service use into four groups: no care, informal care, formal care from a public provider, and formal care from a private provider. This classification aligns with previous research on healthcare facility choice in India and Southeast Asia [16,32]. Under *no care*, we grouped individuals who did not use care in the last 30 days; under *informal care*, we grouped individuals who used care from a private pharmacy, a traditional healer, or by individuals themselves using home remedies; under *formal care public*, we grouped individuals who used care from a subcenter (SC), primary health center (PHC), urban health center (UHC), dispensary, community health center (CHC), mobile medical unit, or public hospital; and under *formal care private*, we grouped individuals who used care from a non-governmental organization or trust clinic/hospital, a private doctor/clinic, or a private hospital. If individuals visited a facility different from those listed above or could not recall the facility type but used care from a formal provider, they were categorized under formal private care. Private pharmacies were classified as informal care due to the frequent lack of formally trained personnel, acceptance of undocumented payments, and sale of unlicensed and unprescribed drugs [33], which are common characteristics of informal providers [34].

**Table 1.** Variables and their measurements.

Variables	Measurements
Outcome variable:	
Health service use in the past 30 days:	0 = No care
	1 = Informal care
	2 = Formal care public
	3 = Formal care private
Explanatory variables:	
*Contextual factors/health system characteristics*	
State groups	0 = HAQ index below Indian average (Bihar, Chhattisgarh, Meghalaya, Uttar Pradesh)
	1 = HAQ index above Indian average (Kerala, Tamil Nadu, Gujarat)
*Predisposing factors*	
Age (years)	Continuous measure
Household size	0 = 1–5
	1 = 6 and above
Being the Household head	0 = No
	1 = Yes
Household head sex	0 = Female
	1 = Male
Individual sex	0 = Female
	1 = Male
Marital status	0 = Not married
	1 = Married
*Enabling factors*	
Household head education level	0 = No education
	1 = Up to primary school
	2 = Secondary school and above
Individual education level	0 = No education
	1 = Up to primary school
	2 = Secondary school and above
Socioeconomic quintile	1 = Q1 (poorest)
	2 = Q2
	3 = Q3
	4 = Q4
	5 = Q5 (least poor)
Enrolled under any publicly funded health insurance scheme	0 = No
	1 = Yes
*Need factors*	
Number of CNCDs	0 = One
	1 = Two or more
Type of CNCD	0 = Minor CNCD
	1 = Major CNCD (cardiovascular diseases, diabetes and chronic kidney disease, cancer, chronic respiratory diseases)
Limitations in daily activities caused byCNCD	0 = No limitations
	1 = Temporary limitations
	2 = Permanent limitations

HAQ index, Health access and quality index; CNCDs, chronic non-communicable diseases; Q1–Q5, quintiles.

We selected explanatory variables that influence healthcare-seeking, based on a literature review, our prior work in other settings [13,16], and the Andersen's behavioral model of health service use. This model conceptualizes healthcare-seeking behavior as influenced by barriers and facilitators, categorized into (i) contextual factors or health system characteristics; (ii) predisposing factors; (iii) enabling factors; and (iv) need factors [35]. To capture healthcare accessibility based on residence, we included the study states as a contextual factor. We grouped states according to the Healthcare Access and Quality (HAQ) index, which assesses the accessibility of effective treatment for various conditions, including CNCDs. States with an HAQ index above the national average included Kerala, Tamil Nadu, and Gujarat, while Bihar, Chhattisgarh, Meghalaya, and Uttar Pradesh fell below the average [36]. We considered age, household size, being the household head, sex (individual and of the household head), and marital status as predisposing factors. As enabling factors, we included education level (of the individual and the household head), socioeconomic quintiles, and health insurance status. We derived socioeconomic quintiles from household per-capita monthly consumption expenditure (including food and non-food expenditures, averaged over 30 days, and household size) as a wealth proxy.

For individuals with chronic health conditions, need factors included the number of chronic conditions, the type of CNCD, and limitations in daily activities caused by the chronic illness. The questionnaire defined chronic illnesses as conditions lasting for more than 3 months. Interviewers used a predefined list of common chronic conditions. Conditions reported by respondents beyond the ones specifically probed for were classified as ‘others’ and ‘other disability’, which we further subsumed into ‘other disability or disease’ (Table 2). To focus on CNCDs, we excluded chronic communicable diseases and nutritional diseases from our disease classification, following the classification of the Global Burden of Disease study [37]. We reclassified the type of CNCD into Major and Minor CNCDs, as commonly referred to by the World Health Organization [38]. Major CNCDs include cardiovascular diseases, chronic respiratory conditions (Asthma/COPD), diabetes, chronic kidney diseases, and cancer, as these account for the highest CNCD-related mortality in India [19]. Minor CNCDs include the remaining diagnoses. High-burden diseases significantly shape prevention and control programs, influencing early diagnosis, treatment, and health literacy and awareness efforts by the World Health Organization [38] and the Government of India [39]. Reclassifying Major and Minor CNCDs, therefore, reflects perceived healthcare needs. We categorized multimorbid individuals, with chronic conditions from both groups, as Major CNCDs, assuming a higher perceived need for healthcare. We assessed limitations in daily activities based on participants' self-reports over the past 30 days, classifying them as ‘no limitations’, ‘temporary limitations’, and ‘permanent limitations’. This classification aligns with nationwide reports like the Indian National Sample Survey Organization Health Round, to ensure comparability [40].

**Table 2.** CNCD reclassification, reported cases, and proportions per CNCD category.

Type of CNCD	Name of CNCD*	N	Percentage (%)
Major CNCDs (*N* = 2110, 41.7%)	Hypertension/high blood pressure	871	16.2
	Diabetes	503	9.4
	Asthma/chronic respiratory failure	344	6.4
	Cancer (any type)	162	3.0
	Chronic heart disease	150	2.8
	Myocardial infarction/heart attack	83	1.5
	Chronic renal/kidney diseases	70	1.3
	Stroke	68	1.3
Minor CNCDs (*N* = 2951, 58.3%)	Other disability or disease	1355	25.5
	Rheumatoid arthritis	524	9.5
	Goiter/thyroid disorder	163	3.0
	Chronic skin disease/psoriasis	158	2.9
	Chronic liver disease	117	2.1
	Mental disorder	113	2.1
	Gall stone/cholecystitis	110	1.3
	Cataract	106	1.9
	Glaucoma	97	1.8
	Piles, anal fissure, anal fistula	97	1.8
	Epilepsy	77	1.4
	Renal stone	72	1.3
	Rheumatic fever/heart disease	42	0.8
	Sinusitis, tonsillitis	39	0.7
	Pyorrhea/chronic periodontitis/gum disease	31	0.6
	Fluorosis	28	0.5
	Total	5380	

*CNCD*, chronic non-communicable disease; *N*, number.

This table shows the total conditions and their proportions as reported by 5061 respondents. The conditions of 5061 respondents add up to 5380 because some people reported more than one condition.

*Interviewers asked the study participants about their chronic illnesses using a list of common chronic conditions. Conditions reported by respondents beyond the ones specifically probed for were classified as ‘others’ and ‘other disability’ by the interviewers, which we further subsumed into ‘other disability or disease’.

### Analytical approach

First, we used descriptive statistics (chi-square tests and t-tests) to analyze explanatory variables, comparing differences in our study sample with the overall survey population, as well as differences between eligible households and households that submitted PM-JAY claims (steps two and three of the data collection process). Then, we assessed associations between explanatory variables and our outcome, health service use, using chi-square tests for categorical variables and ANOVA to investigate differences in group means for continuous variables. Last, we used multilevel multinomial logistic regression to estimate determinants of health service use, accounting for the clustered data structure (individuals nested within households). Additional robust standard errors accounted for intrahousehold correlation. ‘No care’ was defined as a reference group for our dependent variable. A likelihood ratio test confirmed the improved fit of the multilevel model over a single-level model. We specified the variance/covariance matrix as unstructured and used Akaike's Information Criterion (AIC) to determine the best-fitting model, optimize the variance/covariance matrix, and select variables for the final model. A stratified regression model examined differences between eligible households and claim households. All analyses were conducted in Stata 17.0 SE, with statistical significance set at 5%.

### Ethical considerations

The primary PM-JAY evaluation study received ethical approval and written consent from all study participants [31]. Since we received fully anonymized secondary data, we did not need separate ethical approval.

## Results

Among 51,820 individuals aged 15 and older, 5,061 (10%) from 4,220 households reported at least one CNCD. Compared with the entire sample, individuals with a CNCD were older (mean (SD) age 51.3 (±16.3)), more likely to live both in states with a below-average HAQ index (57.4%) and in smaller households (52.8%), were household heads (84.7%), female (24.5%), married (75.5%), less educated (58.1% with no education, 18.6% with secondary school or above), of higher socioeconomic quintiles (19.6% from the highest, 18.6% from the lowest quintile), and enrolled in a PFHI scheme (76.8%). Most individuals reported only one CNCD (94.5%), with Minor CNCDs being the most common (58.3%). Functional limitations in daily activities were prevalent, with 36.4% experiencing temporary and 16.4% daily limitations (Appendix Table S1). The most frequently reported CNCDs were hypertension (*n* = 871, 16.2%), rheumatoid arthritis (*n* = 524, 9.5%), and diabetes (*n* = 503, 9.4%). The least frequent was fluorosis (*n* = 28, 0.5%) (Table 2).

Table 3 reports distributions and bivariate associations between explanatory variables and healthcare utilization. Of 5,061 individuals with a CNCD, 5,046 provided information on health service use: 1,168 (23.1%) had not sought care in the past 30 days, 535 (10.6%) used informal care, while 922 (18.3%) and 2,421 (48.0%) used formal public and private care, respectively. All explanatory variables were significantly associated with healthcare use, and age showed significant mean differences across different care-seeking patterns. Those who did not seek care were more frequently without formal education (60.3%), from lower socioeconomic quintiles (22.2% for the lowest quintile), uninsured (29.3%), suffering from Minor CNCDs (73.4%), and not experiencing limitations from their disease (56.1%). Informal care users were more frequently household heads (54.2%), male (51.6%), and displayed educational trends similar to non-users. Formal public care users were more frequently living in states with an above-average HAQ index (62.3%), female household heads (20.2%), and unmarried (27.5%). Their socioeconomic profile resembled that of non-users, while informal or formal private care users were more frequently from higher socioeconomic quintiles (23.2% and 21.2% for the highest quintile, respectively). Health insurance coverage was higher among formal public (85.2%) and formal private care users (77.2%). Individuals with Major CNCDs or with any form of disease-caused activity limitation were more frequently associated with any health service use other than no care, with the highest proportions observed among formal public care users (56.7% with Major CNCDs, 41.4% with temporary, and 18.7% with permanent limitations).

**Table 3.** Health service use by socio-demographics and chronic conditions.

Variables	No care (*N* = 1168, 23.1%)	Informal care (*N* = 535, 10.6%)	Formal care public (*N* = 922, 18.3%)	Formal care private (*N* = 2421, 48.0%)	Total (5046)	*p* value
*Continuous variables:*	***Mean (SD)***	***Mean (SD)***	***Mean (SD)***	***Mean (SD)***	***Mean (SD)***	
Age (years)	50.1 (±16.9)	52.9 (±15.5)	54.7 (±16.0)	50.2 (±16.2)	51.3 (±16.3)	<0.001
*Categorical variables:*	***N (%)***	***N (%)***	***N (%)***	***N (%)***	***N (%)***	
State groups						<0.001
HAQ index below Indian average	988 (84.6%)	444 (83.0%)	348 (37.7%)	1879 (77.6%)	3659 (72.5%)	
HAQ index above Indian average	180 (15.4%)	91 (17.0%)	574 (62.3%)	542 (22.4%)	1387 (27.5%)	
Household size						<0.001
1–5	650 (55.7%)	301 (56.3%)	619 (67.1%)	1328 (54.9%)	2898 (57.4%)	
6 and above	518 (44.3%)	234 (43.7%)	303 (32.9%)	1093 (45.1%)	2148 (42.6%)	
Being the household head						<0.001
No	632 (54.1%)	245 (45.8%)	435 (47.2%)	1353 (55.9%)	2665 (52.8%)	
Yes	536 (45.9%)	290 (54.2%)	487 (52.8%)	1068 (44.1%)	2381 (47.2%)	
Household head sex						<0.001
Female	175 (15.0%)	71 (13.3%)	186 (20.2%)	338 (14.0%)	770 (15.3%)	
Male	993 (85.0%)	464 (86.7%)	736 (79.8%)	2083 (86.0%)	4276 (84.7%)	
Individual sex						0.006
Female	613 (52.5%)	259 (48.4%)	493 (53.5%)	1360 (56.2%)	2725 (54.0%)	
Male	555 (47.5%)	276 (51.6%)	429 (46.5%)	1061 (43.8%)	2321 (46.0%)	
Marital status						0.017
Not married	303 (25.9%)	119 (22.2%)	254 (27.5%)	558 (23.0%)	1234 (24.5%)	
Married	865 (74.1%)	416 (77.8%)	668 (72.5%)	1863 (77.0%)	3812 (75.5%)	
Household head education level						<0.001
No education	650 (55.7%)	293 (54.8%)	420 (45.6%)	1242 (51.3%)	2605 (51.6%)	
Primary school	300 (25.7%)	134 (25.0%)	349 (37.9%)	614 (25.4%)	1397 (27.7%)	
Secondary school and above	218 (18.7%)	108 (20.2%)	153 (16.6%)	565 (23.3%)	1044 (20.7%)	
Individual education level						<0.001
No education	704 (60.3%)	332 (62.1%)	461 (50.0%)	1430 (59.1%)	2927 (58.0%)	
Up to primary school	253 (21.7%)	125 (23.4%)	290 (31.5%)	509 (21.0%)	1177 (23.3%)	
Up to secondary school and above	211 (18.1%)	78 (14.6%)	171 (18.5%)	482 (19.9%)	942 (18.7%)	
Socioeconomic quintile						<0.001
Q1 (poorest)	259 (22.2%)	71 (13.3%)	198 (21.5%)	410 (16.9%)	938 (18.6%)	
Q2	260 (22.3%)	120 (22.4%)	220 (23.9%)	495 (20.4%)	1095 (21.7%)	
Q3	238 (20.4%)	126 (23.6%)	188 (20.4%)	482 (19.9%)	1034 (20.5%)	
Q4	212 (18.2%)	94 (17.6%)	161 (17.5%)	521 (21.5%)	988 (19.6%)	
Q5 (least poor)	199 (17.0%)	124 (23.2%)	155 (16.8%)	513 (21.2%)	991 (19.6%)	
Enrolled in any publicly funded health insurance scheme						<0.001
No	342 (29.3%)	138 (25.8%)	136 (14.8%)	553 (22.8%)	1169 (23.2%)	
Yes	826 (70.7%)	397 (74.2%)	786 (85.2%)	1868 (77.2%)	3877 (76.8%)	
Number of CNCDs						0.041
One	1120 (95.9%)	510 (95.3%)	860 (93.3%)	2280 (94.2%)	4770 (94.5%)	
Two or more	48 (4.1%)	25 (4.7%)	62 (6.7%)	141 (5.8%)	276 (5.5%)	
Type of CNCD						<0.001
Minor CNCD	857 (73.4%)	324 (60.6%)	399 (43.3%)	1364 (56.3%)	2944 (58.3%)	
Major CNCD (cardiovascular diseases, diabetes and chronic kidney disease, cancer, chronic respiratory diseases)	311 (26.6%)	211 (39.4%)	523 (56.7%)	1057 (43.7%)	2102 (41.7%)	
Limitations in daily activities caused by CNCD						<0.001
No limitations	655 (56.1%)	269 (50.3%)	368 (39.9%)	1088 (44.9%)	2380 (47.2%)	
Temporary limitations	349 (29.9%)	209 (39.1%)	382 (41.4%)	897 (37.1%)	1837 (36.4%)	
Permanent limitations	164 (14.0%)	57 (10.7%)	172 (18.7%)	436 (18.0%)	829 (16.4%)	

SD, standard difference; N, number; HAQ index, Health access and quality index; CNCDs, chronic non-communicable diseases; Q1–Q5, quintiles.

*p* values by ANOVA for continuous variables and chi-square tests for binary/categorical variables.

Table 4 displays the results of the multilevel multinomial logistic regression model, confirming that individuals from higher socioeconomic quintiles (highest quintile: RRR = 4.017, 95% CI [2.13, 7.56]), those with at least one Major CNCD (RRR = 2.293, 95% CI [1.59, 3.30]), and those with temporary limitations in daily activities (RRR = 1.685, 95% CI [1.16, 2.45]) were more likely to seek informal care compared with no care.

**Table 4.** Health service use: estimated coefficients in multilevel multinomial logistic regression model.

	Informal care vs. No care^1^		Formal care public vs. No care		Formal care private vs. No care	
	RRR	[95%-CI]	RRR	[95%-CI]	RRR	[95%-CI]
Age (years)	1.006	[0.99,1.02]	1.010	[1.00,1.02]	0.992	[0.98,1.00]
State groups						
HAQ index below Indian average	1.000		1.000		1.000	
HAQ index above Indian average	0.767	[0.44,1.35]	23.915***	[9.01,63.44]	1.276	[0.88,1.85]
Household size						
1-5	1.000		1.000		1.000	
6 and above	1.392	[0.96,2.02]	0.799	[0.55,1.16]	1.420**	[1.09,1.85]
Being the household head						
No	1.000		1.000		1.000	
Yes	1.616	[0.99,2.63]	1.200	[0.76,1.90]	1.171	[0.84,1.63]
Household head sex						
Female	1.000		1.000		1.000	
Male	1.337	[0.71,2.53]	0.964	[0.54,1.73]	1.186	[0.77,1.83]
Individual sex						
Female	1.000		1.000		1.000	
Male	0.973	[0.59,1.60]	0.765	[0.48,1.22]	0.748	[0.54,1.04]
Marital status						
Not married	1.000		1.000		1.000	
Married	1.192	[0.77,1.84]	1.209	[0.79,1.84]	1.244	[0.93,1.66]
Household head education level						
No education	1.000		1.000		1.000	
Primary school	0.817	[0.49,1.37]	1.179	[0.73,1.91]	0.921	[0.65,1.31]
Secondary school and above	1.327	[0.74,2.37]	0.767	[0.43,1.36]	1.325	[0.90,1.95]
Individual education level						
No education	1.000		1.000		1.000	
Up to primary school	1.097	[0.65,1.84]	1.530	[0.92,2.53]	0.895	[0.63,1.28]
Up to secondary school and above	0.635	[0.34,1.20]	1.951*	[1.06,3.60]	0.975	[0.64,1.48]
Socioeconomic quintile						
Q1 (poorest)	1.000		1.000		1.000	
Q2	2.338**	[1.32,4.14]	1.056	[0.65,1.73]	1.469*	[1.01,2.13]
Q3	3.022***	[1.67,5.47]	0.760	[0.44,1.31]	1.655*	[1.12,2.44]
Q4	2.358**	[1.28,4.35]	0.667	[0.37,1.21]	2.188***	[1.46,3.27]
Q5 (least poor)	4.017***	[2.13,7.56]	0.561	[0.29,1.07]	2.441***	[1.61,3.70]
Enrolled in any publicly funded health insurance scheme						
No	1.000		1.000		1.000	
Yes	1.182	[0.79,1.76]	1.553*	[1.02,2.35]	1.478**	[1.12,1.95]
Type of CNCD						
Minor CNCD	1.000		1.000		1.000	
Major CNCD (cardiovascular diseases, diabetes and chronic kidney disease, cancer, chronic respiratory diseases)	2.293***	[1.59,3.30]	3.836***	[2.61,5.64]	2.911***	[2.23,3.80]
Limitations in daily activities caused by CNCD						
No limitations	1.000		1.000		1.000	
Temporary limitations	1.685**	[1.16,2.45]	2.288***	[1.56,3.35]	1.844***	[1.41,2.41]
Permanent limitations	0.913	[0.53,1.56]	3.448***	[2.09,5.70]	2.413***	[1.69,3.45]
Observations Cluster (Households)	5,046 4,220					

RRR, relative risk ratio; CI, confidence interval; HAQ index, Health access and quality index; CNCDs, chronic non-communicable diseases; Q1–Q5, quintiles.

Significant at ***1%, **5%, and * 10%.

^a^We considered no care as our reference category for multinomial logistic regression.

We also confirmed that residents who were from an above-average HAQ index state (RRR = 23.915, 95% CI [9.01, 63.44]) had an individual education level of up to secondary school and above (RRR = 1.951, 95% CI [1.06, 3.60]) were enrolled in a government health insurance scheme (RRR = 1.553, 95% CI [1.02, 2.35]), suffered from a Major CNCD (RRR = 3.836, 95% CI [2.61, 5.64]), and were experiencing temporary or permanent limitations in daily activities (permanent limitations: RRR = 3.448, 95% CI [2.09, 5.70]) have a higher probability of seeking care at formal public providers in comparison to no care.

Our model further shows that individuals from larger households (RRR = 1.420, 95% CI [1.09, 1.85]) from a higher socioeconomic quintile (highest quintile: RRR = 2.441, 95% CI [1.61, 3.70]), enrolled in a government health insurance scheme (RRR = 1.478, 95% CI [1.12, 1.95]), with a Major CNCD (RRR = 2.911, 95% CI [2.23, 3.80]), and experiencing temporary or permanent limitations in daily activities (permanent limitations: RRR = 2.413, 95% CI [1.69, 3.45]) are more likely to seek care at formal private providers in comparison to no care.

Stratified analyses comparing eligible and claim households are provided in the supplementary materials (Appendix Tables S2–4).

## Discussion

Our study makes an important contribution to the existing literature. We found that 9.8% of the individuals reported having a CNCD, a value that is lower than epidemiological estimates [41], but in line with other studies in India using self-reported data [23]. Our figure is also higher than the findings from other LMICs [42]. The relatively high frequency of rheumatoid arthritis in our study likely reflects self-reporting inaccuracies. Previous research in India indicates a much lower prevalence of rheumatoid arthritis, suggesting that conditions classified as such in our study may instead reflect cases of general arthritis, musculoskeletal conditions, or other chronic joint pain syndromes, which are more common and share prevalence rates similar to our results [43]. While this raises questions about disease awareness and potential reporting bias, it is important to clarify that population-based surveys like ours are designed not to assess disease prevalence but to exclusively capture people's perceptions of illness as the starting point in their health-seeking journey [35]. Thus, we do not directly compare our findings on reported disease frequencies with studies estimating disease burden and prevalence.

Our first key finding is that nearly one-fourth of our sample, 1,168 individuals (23.1%) reported no health service use in the past 30 days. These non-users were less likely to experience disease-related limitations, potentially perceiving their treatment need as low. However, interpreting non-utilization rates requires caution: relying on self-reported data means we cannot assess the objective treatment need, and not all chronic conditions require monthly care, particularly when well-managed. Nonetheless, our findings may also indicate undiagnosed or poorly controlled chronic conditions, potentially contributing to India's rising disease burden and premature mortality [44]. Another study from India on outpatient care at rural primary health centers found similar frequencies of patients not seeking or not receiving care, regardless of the disease type (comparing injuries, non-communicable diseases, and communicable diseases). This suggests that non-utilization in our study may not be entirely explained by longer treatment intervals for chronic conditions [45]. Further research and qualitative inquiry are needed to explain non-utilization for chronic conditions.

Second, our findings reveal distinct patterns in healthcare service use and key factors differentiating users from non-users. Most individuals sought formal care, and among those, almost three-fourths chose private sector services. Private sector dominance in outpatient healthcare utilization in India has been well documented [32,46]. Prior studies suggest that inadequate public sector infrastructure (low staff, equipment, and essential medicine availability) drives patients toward the private sector [44]. Public primary facilities are frequently bypassed [47] when service quality in government facilities is regarded as low or services are unavailable [48]. Those who can afford it often prefer private providers [23,24], despite the higher treatment costs [29]. Our regression results confirm prior evidence [20,23] that socioeconomic status (usually referring to a household's expenditure, income, and/or asset profile) plays a crucial role in healthcare access. Individuals in higher socioeconomic quintiles were significantly more likely to seek formal private care (when compared with no care).

Surprisingly, positive insurance status also increases the likelihood of using formal healthcare in both public and private sectors, even though PM-JAY and earlier PFHIs did not cover outpatient care in most states during data collection. This aligns with previous research, indicating that PFHI schemes in India have contributed to increased outpatient service utilization [49].

Since private healthcare utilization is often associated with higher out-of-pocket expenditure and increased financial hardship, the care preferences observed in our sample may expose individuals to economic vulnerability [50]. In fact, a recent study from India found that outpatient care posed a greater financial burden than hospitalization [51]. Further research is needed to confirm this hypothesis. To improve access and financial protection, policy efforts should strengthen CNCD service provision in the public sector and expand PFHI coverage to include outpatient care for chronic conditions. Some Indian states, such as Tamil Nadu and Karnataka, provide potential models for such reforms. By designating certain procedures for exclusive performance and reimbursement in public facilities, Karnataka saw a rise in scheme beneficiaries receiving treatment in public health facilities between 2018 and 2022 [52,53]. Enhancing public sector capacity and implementing strategic measures to direct and retain as much patient demand as possible within government facilities while referring only complex cases to the private sector [54] ensures efficient use of PFHI resources and prioritizes public sector utilization. Meghalaya's 2021 health Policy [55] introduced coverage for certain outpatient services within its Megha Health Insurance Scheme, the state-specific version of PM-JAY [56,57]. Further research should evaluate the impact of these policies on outpatient healthcare access and financial protection for CNCDs.

Interestingly, informal care use (when compared with no care) resembles formal private care use, with individuals from higher socioeconomic quintiles being more likely to seek it. This might stem from our classification of private pharmacies as informal care. Given that medications constitute a significant portion of outpatient healthcare expenses in India [58], some informal care users in our sample may be wealthier individuals buying medicines directly (either as a preference or out of necessity due to shortages of essential drugs at formal providers) [59]. Qualitative studies suggest that when formal providers are inaccessible, individuals may turn to informal providers or pharmacies, often facing higher costs and lower-quality care [60].

Third, our findings highlight the critical role of a functioning public healthcare system in shaping healthcare-seeking behavior. The strong association between state of residence and health service use reveal significant regional disparities: individuals from above-average HAQ index states (Kerala, Tamil Nadu, and Gujarat) were significantly more likely to use formal public care (when compared with no care). This likely reflects subnational differences in healthcare access and quality, which are among the most pronounced worldwide [36]. Kerala and Tamil Nadu, among India's wealthier states, have a higher density of health workers [61] and a greater availability of government health facilities in rural areas [62], whereas public health expenditure remains particularly low in states such as Bihar and Uttar Pradesh [63]. Ambitious policy initiatives, such as Tamil Nadu's 2023 and 2030 health sector vision, aim to elevate public healthcare delivery to international standards and may be increasing government health facilities use, despite ongoing challenges in the quality of care and health outcomes [29,64]. The proportion of ailments treated in public hospitals varies significantly across states [40], and our findings align with previous reports documenting the heterogeneity in healthcare access and provider choice, which are closely linked to public health spending [65]. Since our study deliberately selected states to reflect these variations in healthcare system performance, these trends were expected, but evidence from our multinomial regression analysis of how these regional differences affect the use of outpatient care is nonetheless valuable.

Our final key finding highlights how disease type and daily activity limitations as need factors affect healthcare utilization. Individuals with Major CNCDs were significantly more likely to seek care across all healthcare options (compared with no care). The conditions classified under Major CNCDs contribute substantially to the disease burden in India and globally [66]. Many of these conditions, such as hypertension and diabetes, are targeted by outreach and screening initiatives, and health literacy efforts, such as the National Programme for Prevention & Control of Cancer, Diabetes, Cardiovascular Diseases & Stroke (NPCDCS) [39]. These efforts may facilitate early diagnosis, referrals, and treatment initiation, thereby increasing awareness and perceived healthcare needs. While evaluations of the NPCDCS (later renamed NP-NCD) have identified implementation gaps and poor performance in certain states [67], our findings suggest that these policies and existing health system structures still influence healthcare utilization. Similar trends of increased reliance on formal healthcare for Major CNCDs have been observed in other LMICs [16,42]. Furthermore, as the duration of daily activity limitations caused by the CNCD increased, so did the likelihood of using health services across all care options (compared with no care). Notably, permanent limitations were only significantly associated with formal care use. These results align with previous studies indicating that individuals with a higher disease burden tend to seek care from formal providers, which are often perceived as offering higher-quality treatment [24]. Another explanation is that low health literacy may hinder prescribed treatment adherence at subclinical stages of the disease [68], leading individuals to seek care only when symptoms affect daily activities [29]. These factors might contribute to the number of untreated or uncontrolled diagnoses and undesirable health outcomes [44].

It is noteworthy that while most explanatory variables showed significant associations with healthcare utilization in the bivariate analysis, only a few remained significant predictors in the multivariable regression model. Beyond illness-based need factors, realized access and healthcare use in our sample were primarily shaped by the state of residence and the household's socioeconomic quintile, along with health insurance status to a lesser extent. Other predisposing and enabling factors lost significance, with only household size and individual education level retaining borderline significance of 10% for some outcome categories.

### Methodological considerations

Our study advances prior research conducted in Bangladesh by the authors [16], and other studies that informed its design and interpretation. A key strength is its large, geographically diverse sample, which is not restricted to a single CNCD, region, or type of healthcare provider, nor limited to facilities from a single level of the health system's hierarchy (primary-, secondary-, or tertiary care). This broad scope distinguishes our work from previous studies. Furthermore, our large sample allowed the use of a more complex, two-level regression model, representing a significant advancement over prior research. By accounting for the hierarchical structure of the clustered data, this model offers a more precise estimation of both individual- and group-level determinants of healthcare-seeking behavior [69]. We surpass the Bangladesh study approach by differentiating between private and public formal care. This adjustment better reflects the pluralistic healthcare landscape in India and the population's complex decision-making processes. These methodological and contextual refinements provide valuable insights into PM-JAY-eligible individuals, who represent approximately 40% of India's population and include some of the country's most disadvantaged households [30]. Understanding the healthcare-seeking behavior of this group is crucial for future evaluations of the scheme's ambitions.

Despite its robust foundation, some limitations may affect our study's findings. First, our sample is primarily rural and consists exclusively of PM-JAY eligible individuals and households. While this provides a targeted analysis of a disadvantaged segment of the population, it limits the study's generalizability. Second, as with all population-based surveys relying on self-reported data, our measure of health service utilization is subject to recall and measurement bias. On the one hand, utilization may be underestimated if participants fail to report all healthcare encounters or if they receive care at longer intervals (>30 days) for well-controlled chronic conditions. However, research indicates that longer recall periods significantly reduce the accuracy of self-reports, whereas shorter recall periods minimize recall bias, particularly for regular outpatient visits [70]. This favorable trade-off enhances reliability when analyzing micro-level data and individual characteristics [71]. On the other hand, because respondents were not subject to medical examination, we could not determine the actual health needs driving healthcare seeking. Hence, we assessed healthcare utilization against illness reporting and not against actual medical needs. Third, we acknowledge that illness reporting bias may have influenced our estimates [50,72]. To address this, we provide a comparative analysis in the Appendix, detailing how the subgroup reporting at least one CNCD differs from the full survey sample. Fourth, an important methodological consideration is the sampling strategy, which included both a random sample of eligible households and a selected sample of claim households. Households with prior hospitalization may be more engaged with the healthcare system and could thus be more likely to seek care. To assess the potential impact of this bias, we conducted a stratified analysis, the results of which are presented in the Appendix. Importantly, our findings (Appendix Tables S2–4) show no significant deviations from the overall trends observed in our primary analysis. Lastly, the COVID-19 pandemic disrupted our sampling process in Kerala, resulting in a slightly smaller sample size for the state and larger-than-intended differences in state-level subsamples. Despite these limitations, we trust that we have taken sufficient precautions not to compromise the internal validity of our findings.

## Conclusions

Our study provides policy-relevant insights into outpatient healthcare-seeking behavior among the rural poor with CNCDs in India's heterogeneous and pluralistic healthcare system. We observed a sizable proportion of individuals not using regular outpatient chronic care. Outpatient health services utilization in this study is predominantly concentrated in the private sector and largely influenced by a household's socioeconomic status. In contrast, public healthcare services are predominantly used in high-performing states with stronger public health infrastructure, highlighting the potential for greater investments and strengthening of the public healthcare system, ideally at all three levels, to redirect demand towards the public sector, thereby reducing health inequities. Furthermore, we observed higher healthcare utilization across all options for certain high-burden CNCDs that are the primary targets of current health literacy, prevention, and control efforts. Expanding health insurance coverage to include outpatient care and essential medications for chronic conditions could further enhance access to adequate treatment. However, further qualitative inquiry is needed to investigate the underlying reasons for non-utilization and identify additional barriers to care.

## Supplementary Material

05 Supplementary materials_clean.docx

## Data Availability

The data that support the findings of this study are available from Deutsche Gesellschaft für Internationale Zusammenarbeit GmbH, but restrictions apply to the availability of these data, which were used under license for the current study, and so are not publicly available. Data are, however, available from the authors upon reasonable request and with permission from Deutsche Gesellschaft für Internationale Zusammenarbeit GmbH.
