# Supplementary material for: Determinants of outpatient healthcare-seeking behaviors among the rural poor affected by chronic conditions in India: a population-based cross-sectional study in seven states
Source: Glob Health Action. 2025 Apr 14;18(1):2480413. doi: 10.1080/16549716.2025.2480413 (PMC11998304; doi:10.1080/16549716.2025.2480413)
Supplement: 05 Supplementary materials_clean.docx [file ZGHA_A_2480413_SM3214.docx]

| **Appendix Table 1: Sociodemographic characteristics, entire sample and CNCD respondents:** | | | | | |
| --- | --- | --- | --- | --- | --- |
| Variables | Non-CNCD sample  (N=46759, 90.2%) | | CNCD sample (N=5061, 9.8%) | Total  (51820) | *p* value |
| *Continuous variables:* | ***Mean (SD)*** | | ***Mean (SD)*** | ***Mean (SD)*** |  |
| Age (years) | 36.6 (± 17.0) | | 51.3 (± 16.3) | 38.0 (± 17.5) | <0.001 |
|  |  |  |  |  |  |
| *Categorical variables:* |  | ***N (%)*** | ***N (%)*** | ***N (%)*** |  |
| State groups |  | |  |  | <0.001 |
| HAQ index below Indian average | 23827 (51.0%) | | 2905 (57.4%) | 26732 (51.6%) |  |
| HAQ index above Indian average | 22932 (49.0%) | | 2156 (42.6%) | 25088 (48.4%) |  |
| Household size |  | |  |  | <0.001 |
| 1-5 | 34308 (73.4%) | | 2674 (52.8%) | 36982 (71.4%) |  |
| 6 and above | 12451 (26.6%) | | 2387 (47.2%) | 14838 (28.6%) |  |
| Being the household head |  | |  |  | 0.090 |
| No | 7573 (16.2%) | | 773 (15.3%) | 8346 (16.1%) |  |
| Yes | 39182 (83.8%) | | 4288 (84.7%) | 43470 (83.9%) |  |
| Household head sex |  | |  |  | <0.001 |
| Female | 23443 (50.1%) | | 2734 (54.0%) | 26177 (50.5%) |  |
| Male | 23316 (49.9%) | | 2327 (46.0%) | 25643 (49.5%) |  |
| Individual sex |  | |  |  | <0.001 |
| Female | 17394 (37.2%) | | 1238 (24.5%) | 18632 (36.0%) |  |
| Male | 29365 (62.8%) | | 3823 (75.5%) | 33188 (64.0%) |  |
| Marital status |  | |  |  | <0.001 |
| Not married | 17394 (37.2%) | | 1238 (24.5%) | 18632 (36.0%) |  |
| Married | 29365 (62.8%) | | 3823 (75.5%) | 33188 (64.0%) |  |
| Household head education level |  | |  |  | 0.007 |
| No education | 25081 (53.6%) | | 2617 (51.7%) | 27698 (53.5%) |  |
| Primary school | 12793 (27.4%) | | 1399 (27.6%) | 14192 (27.4%) |  |
| Secondary school and above | 8881 (19.0%) | | 1045 (20.6%) | 9926 (19.2%) |  |
| Individual education level |  | |  |  | <0.001 |
| No education | 18561 (39.7%) | | 2938 (58.1%) | 21499 (41.5%) |  |
| Up to primary school | 11882 (25.4%) | | 1180 (23.3%) | 13062 (25.2%) |  |
| Up to secondary school and above | 16316 (34.9%) | | 943 (18.6%) | 17259 (33.3%) |  |

| **Appendix Table 1 (continued): Sociodemographic characteristics, entire sample and CNCD respondents:** | | | | |
| --- | --- | --- | --- | --- |
| Variables | Non-CNCD sample  (N=46759, 90.2%) | CNCD sample (N=5061, 9.8%) | Total  (51820) | *p* value |
| *Categorical variables:* | ***N (%)*** | ***N (%)*** | ***N (%)*** |  |
| Socioeconomic quintile |  |  |  | <0.001 |
| Q1 (poorest) | 10299 (22.0%) | 943 (18.6%) | 11242 (21.7%) |  |
| Q2 | 9646 (20.6%) | 1099 (21.7%) | 10745 (20.7%) |  |
| Q3 | 9382 (20.1%) | 1035 (20.5%) | 10417 (20.1%) |  |
| Q4 | 9051 (19.4%) | 990 (19.6%) | 10041 (19.4%) |  |
| Q5 (least poor) | 8381 (17.9%) | 994 (19.6%) | 9375 (18.1%) |  |
| Enrolled in any publicly funded health insurance scheme |  |  |  | <0.001 |
| No | 13538 (29.0%) | 1172 (23.2%) | 14710 (28.4%) |  |
| Yes | 33221 (71.0%) | 3889 (76.8%) | 37110 (71.6%) |  |
| Number of CNCD |  |  |  |  |
| One | . (.%) | 4785 (94.5%) | . (.%) |  |
| Two or more | . (.%) | 276 (5.5%) | . (.%) |  |
| Type of CNCD |  |  |  |  |
| Minor CNCD | . (.%) | 2951 (58.3%) | . (.%) |  |
| Major CNCD (cardiovascular diseases, diabetes and chronic kidney disease, cancer, chronic respiratory diseases) | . (.%) | 2110 (41.7%) | . (.%) |  |
| Limitations in daily activities caused by CNCD |  |  |  |  |
| No limitations | . (.%) | 2386 (47.1%) | . (.%) |  |
| Temporary limitations | . (.%) | 1844 (36.4%) | . (.%) |  |
| Permanent limitations | . (.%) | 831 (16.4%) | . (.%) |  |
| SD, standard difference; N, number; HAQ index, Health access and quality index; CNCDs, chronic non-communicable diseases; Q1-Q5, quintiles  *p* values by t-test for continuous variables and Chi2 test for binary/categorical variables. | | | | |

| **Appendix Table 2: Sociodemographic characteristics, claim households and eligible households:** | | | | |
| --- | --- | --- | --- | --- |
| Variables | Claim households (N=12549, 24.2%) | Eligible households (n=39271, 75.8%) | Total (51820) | *p*-value |
| *Continuous variables* | ***Mean (SD)*** | ***Mean (SD)*** | ***Mean (SD)*** |  |
| Age (years) | 38.6 (± 17.7) | 37.8 (± 17.4) | 38.0 (± 17.5) | <0.001 |
|  |  |  |  |  |
| *Categorical variables* | ***N (%)*** | ***N (%)*** | ***N (%)*** |  |
| Suffering from a CNCD |  |  |  | <0.001 |
| No | 11179 (89.1%) | 35580 (90.6%) | 46759 (90.2%) |  |
| Yes | 1370 (10.9%) | 3691 (9.4%) | 5061 (9.8%) |  |
| State groups |  |  |  | <0.001 |
| HAQ-Index below Indian average | 8074 (64.3%) | 28596 (72.8%) | 36670 (70.8%) |  |
| HAQ-Index above Indian average | 4475 (35.7%) | 10675 (27.2%) | 15150 (29.2%) |  |
| Household size |  |  |  | <0.001 |
| 1-5 | 5949 (47.4%) | 20783 (52.9%) | 26732 (51.6%) |  |
| 6 and above | 6600 (52.6%) | 18488 (47.1%) | 25088 (48.4%) |  |
| Being the household head |  |  |  | <0.001 |
| No | 9247 (73.7%) | 27735 (70.6%) | 36982 (71.4%) |  |
| Yes | 3302 (26.3%) | 11536 (29.4%) | 14838 (28.6%) |  |
| Household head sex |  |  |  | <0.001 |
| Female | 2354 (18.8%) | 5992 (15.3%) | 8346 (16.1%) |  |
| Male | 10192 (81.2%) | 33278 (84.7%) | 43470 (83.9%) |  |
| Individual sex |  |  |  | 0.072 |
| Female | 6427 (51.2%) | 19750 (50.3%) | 26177 (50.5%) |  |
| Male | 6122 (48.8%) | 19521 (49.7%) | 25643 (49.5%) |  |
| Marital status |  |  |  | 0.001 |
| Not married | 4363 (34.8%) | 14269 (36.3%) | 18632 (36.0%) |  |
| Married | 8186 (65.2%) | 25002 (63.7%) | 33188 (64.0%) |  |
| Household head education level |  |  |  | <0.001 |
| No education | 5411 (43.1%) | 22287 (56.8%) | 27698 (53.5%) |  |
| Primary school | 4083 (32.5%) | 10109 (25.7%) | 14192 (27.4%) |  |
| Secondary school and above | 3052 (24.3%) | 6874 (17.5%) | 9926 (19.2%) |  |
|  |  |  |  |  |

| **Appendix Table 2 (continued): Sociodemographic characteristics, claim households and eligible households:** | | | | |
| --- | --- | --- | --- | --- |
| Variables | Claim households (N=12549, 24.2%) | Eligible households (n=39271, 75.8%) | Total (51820) | *p*-value |
| *Categorical variables* | ***N (%)*** | ***N (%)*** | ***N (%)*** |  |
| Individual education level |  |  |  | <0.001 |
| No education | 4081 (32.5%) | 17418 (44.4%) | 21499 (41.5%) |  |
| Up to primary school | 3049 (24.3%) | 10013 (25.5%) | 13062 (25.2%) |  |
| Up to secondary school and above | 5419 (43.2%) | 11840 (30.1%) | 17259 (33.3%) |  |
| Socio-economic quintile |  |  |  | 0.001 |
| Q1 (poorest) | 2783 (22.2%) | 8459 (21.5%) | 11242 (21.7%) |  |
| Q2 | 2470 (19.7%) | 8275 (21.1%) | 10745 (20.7%) |  |
| Q3 | 2476 (19.7%) | 7941 (20.2%) | 10417 (20.1%) |  |
| Q4 | 2442 (19.5%) | 7599 (19.4%) | 10041 (19.4%) |  |
| Q5 (least poor) | 2378 (18.9%) | 6997 (17.8%) | 9375 (18.1%) |  |
| Enrolled in any publicly funded health insurance scheme |  |  |  | 0.038 |
| No | 3471 (27.7%) | 11239 (28.6%) | 14710 (28.4%) |  |
| Yes | 9078 (72.3%) | 28032 (71.4%) | 37110 (71.6%) |  |
| Number of CNCDs |  |  |  | 0.003 |
| One | 1274 (93.0%) | 3511 (95.1%) | 4785 (94.5%) |  |
| Two or more | 96 (7.0%) | 180 (4.9%) | 276 (5.5%) |  |
| Type of CNCD |  |  |  | <0.001 |
| Minor CNCD | 698 (50.9%) | 2253 (61.0%) | 2951 (58.3%) |  |
| Major CNCD (cardiovascular diseases, diabetes and chronic kidney disease, cancer, chronic respiratory diseases) | 672 (49.1%) | 1438 (39.0%) | 2110 (41.7%) |  |
| Limitations in daily activities caused by CNCD |  |  |  | <0.001 |
| No limitations | 561 (40.9%) | 1825 (49.4%) | 2386 (47.1%) |  |
| Temporary limitations | 567 (41.4%) | 1277 (34.6%) | 1844 (36.4%) |  |
| Permanent limitations | 242 (17.7%) | 589 (16.0%) | 831 (16.4%) |  |
| SD, standard difference; N, number; HAQ index, Health access and quality index; CNCDs, chronic non-communicable diseases; Q1-Q5, quintiles  *p* values by t-test for continuous variables and Chi2 test for binary/categorical variables. | | | | |

| **Appendix Table 3: Health service use: estimated coefficients in multilevel multinomial logistic regression model for eligible household subsample** | | | | | | | | |
| --- | --- | --- | --- | --- | --- | --- | --- | --- |
|  | Informal care vs.  No care^1^ | |  | Formal care public vs. No care | |  | Formal care private vs. No care | |
|  | RRR | [95%-CI] |  | RRR | [95%-CI] |  | RRR | [95%-CI] |
| Age (years) | 1.010 | [1.00,1.02] |  | 1.017^*^ | [1.01,1.03] |  | 0.992 | [0.98,1.00] |
| State groups |  |  |  |  |  |  |  |  |
| HAQ-Index below Indian average | 1.000 |  |  | 1.000 |  |  | 1.000 |  |
| HAQ-Index above Indian average | 1.309 | [0.68,2.52] |  | 43.061^***^ | [10.00,185.46] |  | 2.076^**^ | [1.24,3.47] |
| Household size |  |  |  |  |  |  |  |  |
| 1-5 | 1.000 |  |  | 1.000 |  |  | 1.000 |  |
| 6 and above | 1.318 | [0.87,2.00] |  | 0.805 | [0.51,1.27] |  | 1.385^*^ | [1.03,1.87] |
| Being the household head |  |  |  |  |  |  |  |  |
| No | 1.000 |  |  | 1.000 |  |  | 1.000 |  |
| Yes | 1.416 | [0.82,2.44] |  | 1.450 | [0.81,2.59] |  | 1.226 | [0.83,1.81] |
| Household head sex |  |  |  |  |  |  |  |  |
| Female | 1.000 |  |  | 1.000 |  |  | 1.000 |  |
| Male | 1.193 | [0.59,2.43] |  | 0.815 | [0.39,1.69] |  | 1.243 | [0.74,2.09] |
| Individual sex |  |  |  |  |  |  |  |  |
| Female | 1.000 |  |  | 1.000 |  |  | 1.000 |  |
| Male | 1.003 | [0.57,1.76] |  | 0.683 | [0.38,1.23] |  | 0.699 | [0.47,1.04] |
| Marital status |  |  |  |  |  |  |  |  |
| Not married | 1.000 |  |  | 1.000 |  |  | 1.000 |  |
| Married | 1.063 | [0.66,1.72] |  | 1.396 | [0.83,2.34] |  | 1.120 | [0.80,1.57] |
| Household head education level |  |  |  |  |  |  |  |  |
| No education | 1.000 |  |  | 1.000 |  |  | 1.000 |  |
| Primary school | 0.800 | [0.45,1.44] |  | 1.333 | [0.74,2.39] |  | 0.840 | [0.56,1.26] |
| Secondary school and above | 1.125 | [0.59,2.15] |  | 0.873 | [0.43,1.78] |  | 1.313 | [0.84,2.05] |
| Individual education level |  |  |  |  |  |  |  |  |
| No education | 1.000 |  |  | 1.000 |  |  | 1.000 |  |
| Up to primary school | 1.317 | [0.73,2.39] |  | 1.573 | [0.85,2.90] |  | 1.006 | [0.66,1.53] |
| Up to secondary school and above | 1.117 | [0.55,2.27] |  | 1.897 | [0.89,4.06] |  | 1.217 | [0.74,1.99] |

| **Appendix Table 3 (continued): Health service use: estimated coefficients in multilevel multinomial logistic regression model for eligible household subsample** | | | | | | | | |
| --- | --- | --- | --- | --- | --- | --- | --- | --- |
|  | Informal care vs.  No care^1^ | |  | Formal care public vs. No care | |  | Formal care private vs. No care | |
|  | RRR | [95%-CI] |  | RRR | [95%-CI] |  | RRR | [95%-CI] |
| Socioeconomic quintile |  |  |  |  |  |  |  |  |
| Q1 (poorest) | 1.000 |  |  | 1.000 |  |  | 1.000 |  |
| Q2 | 2.635^**^ | [1.39,4.99] |  | 1.225 | [0.68,2.21] |  | 1.574^*^ | [1.03,2.40] |
| Q3 | 3.094^***^ | [1.61,5.96] |  | 1.083 | [0.58,2.03] |  | 1.666^*^ | [1.08,2.58] |
| Q4 | 2.241^*^ | [1.14,4.41] |  | 0.939 | [0.48,1.86] |  | 2.171^***^ | [1.38,3.42] |
| Q5 (least poor) | 3.784^***^ | [1.88,7.60] |  | 0.626 | [0.29,1.34] |  | 2.293^***^ | [1.44,3.66] |
| Enrolled in any publicly funded health insurance scheme |  |  |  |  |  |  |  |  |
| No | 1.000 |  |  | 1.000 |  |  | 1.000 |  |
| Yes | 1.145 | [0.74,1.76] |  | 1.521 | [0.94,2.45] |  | 1.789^***^ | [1.32,2.42] |
| Type of CNCD |  |  |  |  |  |  |  |  |
| Minor CNCD | 1.000 |  |  | 1.000 |  |  | 1.000 |  |
| Major CNCD (cardiovascular diseases, diabetes and chronic kidney disease, cancer, chronic respiratory diseases) | 2.196^***^ | [1.44,3.35] |  | 4.094^***^ | [2.45,6.83] |  | 3.267^***^ | [2.37,4.51] |
| Limitations in daily activities caused by CNCD |  |  |  |  |  |  |  |  |
| No limitations | 1.000 |  |  | 1.000 |  |  | 1.000 |  |
| Temporary limitations | 2.691^***^ | [1.75,4.15] |  | 1.905^**^ | [1.23,2.96] |  | 2.363^***^ | [1.71,3.26] |
| Permanent limitations | 0.740 | [0.40,1.36] |  | 2.555^**^ | [1.43,4.55] |  | 2.080^***^ | [1.41,3.08] |
| Observations | 3682 |  |  |  |  |  |  |  |
| Cluster (Households) | 3086 |  |  |  |  |  |  |  |

RRR, relative risk ratio; CI, confidence interval; HAQ index, Health access and quality index; CNCDs, chronic non-communicable diseases; Q1-Q5, quintiles
Significant at ***1%, **5% and *10%
^1^We considered no care as our reference category for multinomial logistic regression

| **Appendix Table 4: Health service use: estimated coefficients in multilevel multinomial logistic regression model for claim household subsample** | | | | | | | | |
| --- | --- | --- | --- | --- | --- | --- | --- | --- |
|  | Informal care vs.  No care^1^ | |  | Formal care public vs. No care | |  | Formal care private vs. No care | |
|  | RRR | [95%-CI] |  | RRR | [95%-CI] |  | RRR | [95%-CI] |
| Age (years) | 0.994 | [0.97,1.02] |  | 0.999 | [0.98,1.02] |  | 0.997 | [0.98,1.02] |
| State groups |  |  |  |  |  |  |  |  |
| HAQ-Index below Indian average | 1.000 |  |  | 1.000 |  |  | 1.000 |  |
| HAQ-Index above Indian average | 0.850 | [0.22,3.26] |  | 9.904^***^ | [3.67,26.73] |  | 1.199 | [0.56,2.55] |
| Household size |  |  |  |  |  |  |  |  |
| 1-5 | 1.000 |  |  | 1.000 |  |  | 1.000 |  |
| 6 and above | 2.080 | [0.86,5.06] |  | 0.907 | [0.44,1.86] |  | 1.758 | [0.98,3.17] |
| Being the household sex |  |  |  |  |  |  |  |  |
| No | 1.000 |  |  | 1.000 |  |  | 1.000 |  |
| Yes | 2.697 | [0.86,8.46] |  | 0.960 | [0.42,2.21] |  | 1.022 | [0.52,2.02] |
| Household head sex |  |  |  |  |  |  |  |  |
| Female | 1.000 |  |  | 1.000 |  |  | 1.000 |  |
| Male | 1.692 | [0.37,7.70] |  | 1.239 | [0.43,3.55] |  | 0.858 | [0.37,2.01] |
| Individual sex |  |  |  |  |  |  |  |  |
| Female | 1.000 |  |  | 1.000 |  |  | 1.000 |  |
| Male | 0.660 | [0.21,2.08] |  | 0.867 | [0.39,1.95] |  | 0.764 | [0.40,1.46] |
| Marital status |  |  |  |  |  |  |  |  |
| Not married | 1.000 |  |  | 1.000 |  |  | 1.000 |  |
| Married | 2.171 | [0.70,6.75] |  | 1.270 | [0.56,2.90] |  | 2.051^*^ | [1.06,3.96] |
| Household head education level |  |  |  |  |  |  |  |  |
| No education | 1.000 |  |  | 1.000 |  |  | 1.000 |  |
| Primary school | 0.876 | [0.27,2.85] |  | 1.142 | [0.45,2.90] |  | 1.368 | [0.64,2.93] |
| Secondary school and above | 2.655 | [0.63,11.14] |  | 0.986 | [0.34,2.82] |  | 1.821 | [0.78,4.27] |
| Individual education level |  |  |  |  |  |  |  |  |
| No education | 1.000 |  |  | 1.000 |  |  | 1.000 |  |
| Up to primary school | 1.104 | [0.35,3.48] |  | 1.440 | [0.57,3.63] |  | 0.942 | [0.44,2.00] |
| Up to secondary school and above | 0.239 | [0.05,1.21] |  | 1.378 | [0.47,4.02] |  | 0.867 | [0.36,2.11] |

| **Appendix Table 4 (continued): Health service use: estimated coefficients in multilevel multinomial logistic regression model for claim household subsample** | | | | | | | | |
| --- | --- | --- | --- | --- | --- | --- | --- | --- |
|  | Informal care vs.  No care^1^ | |  | Formal care public vs. No care | |  | Formal care private vs. No care | |
|  | RRR | [95%-CI] |  | RRR | [95%-CI] |  | RRR | [95%-CI] |
| Socioeconomic quintile |  |  |  |  |  |  |  |  |
| Q1 (poorest) | 1.000 |  |  | 1.000 |  |  | 1.000 |  |
| Q2 | 1.369 | [0.36,5.23] |  | 0.876 | [0.33,2.31] |  | 1.129 | [0.49,2.60] |
| Q3 | 1.607 | [0.38,6.71] |  | 0.414 | [0.14,1.20] |  | 1.442 | [0.61,3.43] |
| Q4 | 1.640 | [0.37,7.26] |  | 0.416 | [0.14,1.27] |  | 1.772 | [0.72,4.35] |
| Q5 (least poor) | 2.982 | [0.68,13.16] |  | 0.515 | [0.17,1.57] |  | 2.211 | [0.90,5.43] |
| Enrolled in any publicly funded health insurance scheme |  |  |  |  |  |  |  |  |
| No | 1.000 |  |  | 1.000 |  |  | 1.000 |  |
| Yes | 1.735 | [0.50,5.98] |  | 1.430 | [0.52,3.95] |  | 0.792 | [0.36,1.72] |
| Type of CNCD |  |  |  |  |  |  |  |  |
| Minor CNCD | 1.000 |  |  | 1.000 |  |  | 1.000 |  |
| Major CNCD (cardiovascular diseases, diabetes and chronic kidney disease, cancer, chronic respiratory diseases) | 2.708^*^ | [1.16,6.33] |  | 3.811^***^ | [1.91,7.58] |  | 2.404^**^ | [1.38,4.17] |
| Limitations in daily activities caused by CNCD |  |  |  |  |  |  |  |  |
| No limitations | 1.000 |  |  | 1.000 |  |  | 1.000 |  |
| Temporary limitations | 0.699 | [0.25,1.97] |  | 3.749^**^ | [1.65,8.54] |  | 1.320 | [0.71,2.47] |
| Permanent limitations | 2.969 | [0.78,11.37] |  | 12.934^***^ | [3.82,43.81] |  | 6.175^***^ | [2.41,15.82] |
| Observations | 1364 |  |  |  |  |  |  |  |
| Cluster (Households) | 1134 |  |  |  |  |  |  |  |

RRR, relative risk ratio; CI, confidence interval; HAQ index, Health access and quality index; CNCDs, chronic non-communicable diseases; Q1-Q5, quintiles
Significant at ***1%, **5% and *10%
^1^We considered no care as our reference category for multinomial logistic regression
